# Advances in Bioactive Compounds from Plants and Their Applications in Alzheimer’s Disease

**DOI:** 10.3390/biom16010007

**Published:** 2025-12-19

**Authors:** Steve Pavlov, Santosh Kumar Prajapati, Dhananjay Yadav, Andrea Marcano-Rodriguez, Hariom Yadav, Shalini Jain

**Affiliations:** 1 USF Center for Microbiome Research, Microbiomes Institute, University of South Florida Morsani College of Medicine, Tampa, FL 33613, USA; stevepavlov@usf.edu (S.P.); prajapati11@usf.edu (S.K.P.); dhananjay11@usf.edu (D.Y.); acm11@usf.edu (A.M.-R.); hyadav@usf.edu (H.Y.); 2 Center of Excellence in Aging and Brain Repair, Department of Neurosurgery, Brain and Spine, University of South Florida Morsani College of Medicine, Tampa, FL 33606, USA; 3 Department of Neurosurgery, Brain and Spine; Faculty of Center of Excellence for Aging and Brain Repair, University of South Florida, 12901 Bruce B Downs, MDC78, Tampa, FL 33612, USA

**Keywords:** Alzheimer’s disease, phytochemicals, neuroprotection, multi-target therapy, oxidative stress, gut–brain axis, blood–brain barrier, neuroinflammation

## Abstract

Alzheimer’s disease (AD), the leading cause of dementia worldwide, is characterized by progressive neuronal loss, amyloid-β (Aβ) aggregation, tau hyperphosphorylation, oxidative stress, neuroinflammation, cholinergic dysfunction, and gut–brain axis dysregulation. Despite advances in anti-amyloid therapeutics, current interventions provide only modest symptomatic relief and face limitations in accessibility, cost, and long-term efficacy. Plant-derived bioactive compounds, rooted in traditional medicine systems such as Ayurveda and Traditional Chinese Medicine, have gained increasing attention as multi-target therapeutic agents due to their pleiotropic actions, relative safety, and ability to cross the blood–brain barrier. This review synthesizes mechanistic and translational evidence on major phytochemicals, including withanolides (*Withania somnifera*), curcumin (*Curcuma longa*), ginkgolides and bilobalide (*Ginkgo biloba*), bacosides (*Bacopa monnieri*), ginsenosides (*Panax ginseng*), crocin/safranal (*Crocus sativus*), epigallocatechin-3-gallate (*Camellia sinensis*), rosmarinic acid (*Salvia officinalis*, *Melissa officinalis*), and asiaticosides (*Centella asiatica*). These compounds exert neuroprotective effects by inhibiting Aβ aggregation, reducing tau phosphorylation, scavenging reactive oxygen species, attenuating NF-κB-mediated inflammation, modulating cholinergic signaling, enhancing synaptic plasticity via brain-derived neurotrophic factor/cAMP response element-binding protein (BDNF/CREB) activation, and regulating gut microbiota. Multi-target approach analyses underscore their synergistic potential in targeting interconnected AD pathways. However, translation remains hindered by poor oral bioavailability, rapid metabolism, and variability in clinical outcomes. Advances in delivery platforms, including liposomes, bilosomes, solid lipid nanoparticles, and nanostructured lipid carriers, are improving stability, blood–brain penetration, and therapeutic efficacy in preclinical models. Collectively, plant-derived phytochemicals serve as promising, affordable, and multi-modal candidates for reshaping AD management, bridging traditional knowledge with modern therapeutic innovation.

## 1. Introduction

The main cause of dementia in elderly populations is Alzheimer’s disease (AD), which leads to permanent neuronal decay that results in declining abilities and loss of self-sufficiency [1,2]. Current understanding of AD extends from amyloid-β (Aβ) plaques and neurofibrillary tangles and is now recognized as a complex neuroimmune–metabolic disorder; this combines amyloid and tau pathologies with ongoing neuroinflammation, mitochondrial dysfunction, impaired proteostasis, synaptic breakdown, cerebrovascular damage, and blood–brain barrier disruption [1,2,3,4,5]. The brain regions that control learning and memory functions—including the hippocampus, entorhinal cortex, and mesial temporal lobe—experience these processes, while peripheral systems influence them through the microbiota–gut–brain axis [2,3,4,6,7]. The convergence of gene expression with metabolite and immune and microbial pathways in multi-omics studies demonstrates the requirement for therapeutic approaches that target multiple disease mechanisms instead of focusing on single lesions [5,6,7]. The worldwide incidence of AD keeps increasing because of the growth in our aging population. Current projections show that AD prevalence, mortality rates, and disability numbers will increase substantially among adults aged 60 and older before 2050 unless researchers develop effective disease-modifying treatments [2,8]. The combination of advancing age with genetic predisposition, and lifestyle choices determines an individual’s risk level. The ApoE ε4 allele stands as the leading genetic risk factor for late-onset AD, but rare autosomal–dominant mutations in amyloid precursor protein (APP) and PSEN1 and PSEN2 genes cause early-onset familial AD [1]. The combination of vascular and metabolic diseases with environmental injuries leads to neuroinflammatory and oxidative stress responses, which damage synaptic plasticity and neural resilience [2,5].

The current pharmacological treatments for AD fail to provide sufficient benefits to patients, most likely due to their single-mechanism interaction. The available symptomatic treatments, including cholinesterase inhibitors and memantine, help some patients maintain their cognitive function, but they do not stop the disease from progressing [1,2]. New anti-amyloid antibodies, lecanemab and donanemab, show promise by decreasing amyloid levels and slowing disease progression in specific patient groups, although their broad population benefits remain limited because of high costs, restricted access, and safety concerns [2]. The complex and multifactorial nature of AD biology requires the development of new, safe, and affordable treatments that can treat multiple targets for broad population use at early disease stages. The combination of traditional medical knowledge with contemporary neuropharmacological research has led to the identification of plant-derived bioactive compounds as effective candidates for AD prevention and adjunctive treatments [9,10,11]. The various phytochemical groups, including polyphenols, terpenoids, and alkaloids, demonstrate multiple effects that target different aspects of AD development. The compounds activate Nrf2 to defend against oxidative stress and support mitochondria; they suppress NF-κB and COX-2 to reduce neuroinflammation; they affect amyloid processing through β-secretase (BACE1), Glycogen Synthase Kinase 3 beta (GSK-3β), and α-secretase (ADAM10); they support synaptic health through brain-derived neurotrophic factor/cAMP response element-binding protein (BDNF/CREB) signaling; and they fix the gut–brain equilibrium to decrease inflammation and protect the blood–brain barrier [5,6,7,9,10,11,12,13,14,15,16,17,18,19,20]. Research studies demonstrate that people who consume plant-based diets and flavonoids experience lower risks of developing dementia and cognitive decline [11,12,21]. The development of new formulations and delivery methods for these compounds faces challenges because of their poor bioavailability, unstable pharmacokinetics, and inconsistent quality standards [22,23,24,25,26,27,28,29,30,31,32,33]. Previous reviews have focused on individual herbs and their specific mechanisms of action. This review presents a unified network–pharmacology framework that connects mechanistic pathways to preclinical results, clinical findings, and delivery advancements for major medicinal plants used against AD, including *Withania somnifera*, *Curcuma longa*, Ginkgo biloba, *Bacopa monnieri*, *Panax ginseng*, *Crocus sativus*, *Camellia sinensis*, Salvia officinalis, *Melissa officinalis*, and *Centella asiatica* [9,10,11,13,14,15,16,17,18,19,34,35,36,37,38,39,40,41,42,43,44,45,46,47,48,49,50,51,52,53,54,55,56,57,58,59,60,61,62,63,64,65,66,67,68].

This review demonstrates how these plants target multiple disease pathways, which include amyloid aggregation, tau hyperphosphorylation, neuroinflammation, oxidative stress, cholinergic decline, synaptic breakdown, and gut–brain imbalances in AD. It presents evidence from animal studies to human research, and explains methods to enhance bioavailability and delivery for clinical applications. In addition, this review unites traditional medical practices with modern systems biology to establish phytochemicals as effective polypharmacological treatments that can enhance current treatments for AD prevention and treatment [7,9,10,11,22,23,24,25,69].

## 2. Methods

Search Strategy: To conduct a comprehensive review of bioactive compounds from plants and their effects on Alzheimer’s disease, searches were carried out between 1 July 2025 and 1 November 2025, prioritizing articles published in the last 5 years but also including foundational experiments from the early 2000s. Databases include PubMed, Scopus, Embase, and Google Scholar. Search terms included “Alzheimer’s Disease” or “bioavailability” in combination with “Withania somnifera”, “Curcuma longa”, “ginkgo biloba”, “Bacopa monnieri”, “Panax ginseng”, “Crocus sativus”, “Camellia sinensis”, “salvia officinalis”, “Melissa officinalis”, and “Centella asiatica”. Reference lists of articles found by this search were reviewed and selected based on relevance.

## 3. Plant-Derived Bioactive Compounds in the Management of AD

The etiology of AD includes Aβ aggregation, tau hyperphosphorylation, oxidative stress, neuroinflammation, cholinergic deficits, and gut–brain axis dysregulation; this necessitates therapeutic strategies that engage multiple targets simultaneously [1,5,6]. Plant-derived bioactive compounds that originate from old traditional medicine systems, such as Ayurveda and Traditional Chinese Medicine, have shown promising effects for AD prevention and management [9,10]. These phytochemicals—including polyphenols, terpenoids, and alkaloids—exhibit multiple effects such as scavenging reactive oxygen species (ROS), modulating neuroinflammatory cascades, and regulating gut microbiota to influence central nervous system (CNS) homeostasis [7,11]. In the Framingham Offspring Cohort, long-term flavonoid intake has been linked to a 42–76% reduced risk of AD and related dementias [12]. However, preclinical models demonstrate their ability to cross the BBB, albeit with variable bioavailability [10,70]. This section breaks down the major classes of these compounds, their representative sources, chemical properties, and foundational evidence. These phytochemicals—encompassing polyphenols, terpenoids, and alkaloids—are summarized in Table 1, which outlines their key compounds and plant sources.

**Table 1:** Mechanistic targets and neuroprotective outcomes of key phytochemicals in different models of AD.

Phytochemicals	Targeted Mechanisms	Models	Key Outcomes
Curcumin	GSK-3β/CDK5 suppression, Aβ binding, ADAM10 boosting, tau kinase inhibition. NF-κB/AMPK modulation.	In vitro assays, scopolamine rats. Cell lines, AD rodent models. Streptozotocin rats, microglia cultures.	Reduced Aβ aggregation, improved cognition. Prevented fibril formation, curtailed tau hyperphosphorylation [13,63,71,72,73,74,75].
EGCG	BACE1 suppression, Aβ oligomer remodeling, APP processing shift, cytokine inhibition, Nrf2 activation.	STZ-AD rats, in vitro assays, hippocampal tissues, STZ rats.	Aβ inhibition, cognitive improvement, 20–40% ROS decrease, mitochondrial restoration [15,18,19,70].
Rosmarinic Acid	Aβ binding, fibril disruption, Aβ oligomerization suppression, aggregate remodeling, AChE inhibition, monoamine modulation.	In vitro assays, AD rodents, Aβ cell lines, rodent impairment models, cortical neurons.	Inhibited aggregation, improved memory, elevated acetylcholine [16,17,54,55,56].
Carnosic Acid	COX-2/NF-κB inhibition, Nrf2 activation, ROS scavenging.	Microglial cultures, rodent models.	ROS reduction, cytokine suppression [16].
Withaferin A	Aβ suppression, NF-κB inhibition, oxidative stress reduction, BDNF/CREB activation.	PC-12 cells, rats, rat cortical neurons.	Reduced Aβ accumulation, protected from cytotoxicity, axon/dendrite outgrowth, restored memory deficits [9,35,76,77,78,79].
Withanoside IV/Sominone/Withanolides	Aβ clearance, neuroinflammation reduction. AChE inhibition, cytokine attenuation.	Aβ-injected mice, 5XFAD mice, Wistar rats, SH-SY5Y cells.	Improved memory, prevented neuronal loss, alleviated cognitive dysfunction [9,35,77,80].
Withanamides A/C	Aβ fibril prevention, BBB permeability.	PC-12 cells.	Protected from Aβ-induced death [9,35,76].
Ginkgolides A/B/C/J	BACE1 reduction, cholesterol lowering, Aβ aggregation inhibition.	APP/PS1 mice, PC-12 cells.	Aβ reduction, improved cognition [36,39].
Bilobalide	Membrane stabilization, ROS scavenging, mitochondrial protection.	Streptozotocin rats, Aβ-injected mice.	ROS decrease, neuronal preservation [39].
EGb 761 Extract	NF-κB suppression, antioxidant upregulation, synaptic modulation.	Aged rodents, AD models.	ROS decrease, restored blood flow [34,36,39].
Bacosides A/B	BACE1 inhibition, Aβ binding, and fibril conversion. AChE inhibition, CREB/BDNF activation, synaptic enhancement.	APP cells, scopolamine rats. Aged rodents, PC-12 cells.	Aβ inhibition, memory reversal. Increased dendritic branching, restored cognition [37,40,41].
Saponins	ROS scavenging, cytokine suppression, antioxidant upregulation.	Streptozotocin rats, neuronal cultures.	Normalized peroxidation, reduced IL-6/TNF-α [37,49].
Ginsenosides Rb1/Rg1	Aβ clearance enhancement, tau phosphorylation inhibition via PI3K/Akt, AChE inhibition, BDNF promotion, receptor agonism.	APP/PS1 mice, streptozotocin rats, rodent models.	Reduced phosphorylated tau, improved cognition, elevated acetylcholine, enhanced neurogenesis [44,45,46,47].
Compound K	NF-κB suppression, Nrf2 activation, ROS scavenging.	AD rat models.	ROS reduction, anti-inflammatory shift [44,45].
Crocin/Safranal/Trans-crocetin	Monoamine modulation, AChE inhibition, BDNF/CREB promotion. Aβ suppression, tau inhibition, NF-κB suppression, gut barrier enhancement.	Streptozotocin rats, neuronal cultures, scopolamine rats, microbiota models.	Attenuated fibril formation, cytokine reduction, elevated acetylcholine, enhanced synaptic density, improved learning, decreased BBB permeability [51,58,59].
Madecassoside	ROS scavenging, cytokine suppression, Nrf2 activation.	Aged mice, neurodegenerative models.	ROS normalization, reduced inflammation [38,42,43,57].
Triterpenoids	AChE inhibition, BDNF/CREB promotion, synaptic density enhancement.	Cortical neurons, AD models.	Increased dendritic integrity, neurogenesis [38,42,43,57].
Asiaticoside	Aβ attenuation.	5XFAD mice, neuronal cultures.	Plaque formation inhibition, improved cognition [42,43].
Theanine (with catechins)	Free radical scavenging, metabolic modulation.	Rodent models.	Neuroprotective effects [15,18,19].

**Table 2:** Major AD pathologies, associated molecular targets, and phytochemical interventions.

Pathology	Key Features	Molecular Targets	Key Compounds
Amyloid Cascade	Aberrant APP cleavage by β- and γ-secretase.	BACE1, GSK-3β, ADAM10	Curcumin, Bacosides, EGCG, Withanolide A [13,15,18,19,37,40,41,49,71,75,81].
Oxidative Stress	Reduced cytochrome-c oxidase, ROS elevation.	Nrf2, SOD, GPx, electron transport chain	Withaferin A, Rosmarinic Acid, EGCG [9,15,16,17,18,19,35,52,53,76,77,78].
Neuroinflammation	Microglial/astrocyte activation, cytokine release.	NF-κB, inflammasomes, IL-1β/TNF-α/IL-6	Curcumin, Safranal, Bilobalide [13,14,34,36,39,50,51,71,75,81].
Cholinergic Dysfunction	Loss of nucleus basalis neurons, acetylcholine depletion.	AChE, ChAT, muscarinic/nicotinic receptors	Bacosides, Rosmarinic Acid, Ginsenosides [16,17,37,41,45,46,49,52,53,54,55,60,61,82,83].
Impaired Synaptic Plasticity	Loss of synaptic density and connectivity. Reduced dendritic branching and spine density.	BDNF, CREB	Crocin, Asiaticoside, Theanine [14,15,18,19,38,42,50,51,57].
Tau Pathology	Hyperphosphorylation and NFT formation.	GSK-3β, CDK5, PI3K/Akt	Curcumin, Ginsenosides, Crocin [13,14,46,47,51,63,71,75].
Gut–Brain Axis Dysregulation	Dysbiosis, barrier permeability.	SCFA production, vagal signaling, BBB	Curcumin, Ginsenosides, Crocin, Withanolides [35,44,76,80,84].

*Polyphenols (Including Flavonoids)*: A diverse class of aromatic compounds with multiple hydroxyl groups, abundant in fruits, vegetables, and herbs [7,12]. In addition, polyphenols’ catechol moiety enhances BACE1 inhibition and increases ROS scavenging ability, while polymerization, conjugation, and glycosylation determine its solubility and play a role in BBB penetration [85]. Studies have reported primarily antioxidant and anti-inflammatory effects against oxidative stress, while modulation of the gut–brain axis mitigates systemic inflammation [6,12]. Additionally, preclinical and clinical reports show neuroprotective effects from polyphenols, such as epigallocatechin-3-gallate (EGCG) from *Camellia sinensis* (green tea), curcumin from *Curcuma longa* (turmeric), rosmarinic acid from *Salvia officinalis* (sage), *Melissa officinalis* (lemon balm), alongside other compounds like crocin/safranal from *Crocus sativus* (saffron) [7,12,13,14,15,16,17].

Polyphenols scavenge for ROS and upregulate antioxidant enzymes via Nrf2 pathway activation [7,12]. In addition to antioxidant effects, they can increase acetylcholine concentrations; for instance, Biasibetti et al., (2013) showed that EGCG reverses oxidative stress and reduces acetylcholinesterase (AChE) activity [18]. Polyphenols like EGCG cross the BBB, but at low efficiency (e.g., ~0.1% in plasma and 0.003% in brain tissue in rats). Despite low concentrations, they still inhibit Aβ production and reduce neurotoxicity [15,19,70]. Another polyphenol is curcumin, and it similarly disrupts Aβ aggregation in vitro and in vivo, improving cognitive performance in scopolamine-treated rats [20,63,72,74]. Besides direct CNS interaction, polyphenols also influence gut microbiota by promoting the production of short-chain fatty acids (SCFAs), such as butyrate, which enhance gut barrier integrity; this, in turn, reduces peripheral inflammation and modulates vagal signaling to attenuate CNS amyloid pathology [6,80,86]. Meta-analyses show that diets richer in polyphenols correlate with slower cognitive decline in aging cohorts [12,21]. However, challenges like rapid hepatic metabolism and poor BBB permeability highlight the need for new forms of delivery like nanoformulations, as discussed in Section 6 [22,23,24,25].

*Terpenoids:* Terpenoids are mainly composed of isoprene units, with each derivative terpenoid differing in quantity, while the functional groups determine BBB permeability and aid in AChE binding [87]. Terpenoids counter AD’s components through antioxidants, anticholinesterase, and vasodilatory actions [7]. Key examples include ginkgolides and bilobalide from *Ginkgo biloba*; withanolides (e.g., withaferin A and withanolide A) from *Withania somnifera* (ashwagandha); bacosides A/B from *Bacopa monnieri*; ginsenosides from *Panax ginseng*; and asiaticoside/madecassoside from *Centella asiatica* [9,34,35,36,37,38]. They scavenge for ROS while enhancing cerebral blood flow and mitigating Aβ-induced neurotoxicity [7]. Withanolides stabilize mitochondrial function, which reduces oxidative damage not only by scavenging ROS but also by promoting dendrite outgrowth [35,76,77,78]. In preclinical models, ginkgolides inhibit Aβ aggregation and improve synaptic plasticity [36,39]. Preclinical models with bacosides display the conversion of toxic oligomers into benign structures while reversing memory deficits in AD rodents [37,40,41]. Asiaticosides inhibit BACE1, which is a key enzyme in the production of Aβ, and enhance synaptic density via BDNF upregulation [38,42,43]. Additionally, terpenoids modulate gut dysbiosis with ginsenosides undergoing microbial transformation, which supports systemic bioavailability [84]. Preclinical evidence indicates strong efficacy, with terpenoid-rich extracts alleviating AD symptoms in multiple models, yet interindividual metabolic variability poses a key barrier to clinical translation [10,11].

*Alkaloids:* Alkaloids are nitrogen-containing heterocyclic structures, and the positively charged nitrogen gives them the potential to bind to AChE sites, while some indole alkaloids are more selective towards butyrylcholinesterase [88]. Studies have shown that alkaloid-containing plants like *Panax ginseng* can modulate muscarinic and nicotinic receptors while reducing AChE activity to increase cholinergic transmission [44,45]. Ginsenosides (e.g., Rb1 and Rg1) function similarly to alkaloids, acting as AChE inhibitors and dopaminergic modulators, while reducing tau phosphorylation by regulating PI3K/Akt signaling [44,45,46,47]. In addition, they suppress microglial activation, scavenge ROS, and enhance Aβ clearance in AD models. Their microbial metabolites, such as compound K, amplify gut–brain effects by restoring microbiota balance [80,84,89]. Broader phytochemical properties include anti-aggregation effects on α-synuclein, relevant to AD comorbidities [7]. Together, alkaloids complement polyphenols and terpenoids, contributing synergistic cholinergic enhancement without the side effects of synthetic inhibitors like donepezil [10,82].

Differentiating Crude Extracts and Purified Compounds: Crude plant extracts and purified compounds are often used in studies for AD, but they differ in composition, reproducibility, and dose response. Crude extracts are obtained through solvents like water or methanol that contain a heterogeneous mixture of the bioactive compound. In contrast, a purified compound is isolated with techniques like chromatography and represents a single molecule rather than the whole collection of molecules within a plant. This makes reproducibility far more likely, and the data that is obtained through purified compounds can be more easily explained by chemical interaction. However, it seems that crude extracts can be more effective, potentially due to the multitude of other compounds that are working together synergistically [90].

## 4. Phytochemicals’ Mechanisms of Action

Bioactive compounds from various medicinal plants act through multiple mechanisms to counter AD pathology. They inhibit the amyloid cascade by preventing toxic β-amyloid aggregation (e.g., curcumin from *Curcuma longa*), reduce tau hyperphosphorylation to limit neurofibrillary tangle formation, and improve cholinergic function by enhancing acetylcholine levels and synaptic activity [1,5]. Many compounds also suppress neuroinflammation by downregulating pro-inflammatory cytokines (IL-1β, TNF-α, and IL-6), protect against mitochondrial dysfunction and oxidative stress, and restore gut–brain axis homeostasis to reduce systemic and CNS inflammation [5,6]. Several plants such as *Withania somnifera* (Ashwagandha), *Bacopa monnieri* (Brahmi), *Ginkgo biloba*, and *Curcuma longa* have shown significant neuroprotective, antioxidant, and anti-amyloid potential. We discuss these plants and their mechanisms in greater detail in the following sections and in Table 2. In addition, in vitro studies show robust antioxidant effects, while in vivo results vary significantly; this is the beginning of the translational hurdle, most likely occurring due to the low BBB penetration of these compounds and high volatility during metabolism.

*Withania somnifera (Ashwagandha): Withania somnifera*, commonly known as Ashwagandha, exerts multifaceted neuroprotective effects in AD through its primary bioactive constituents: the withanolides (e.g., withaferin A, withanolide A, and withanoside IV) [9,35,76,77]. In preclinical models, these steroidal lactones have been shown to modulate key enzyme activities and signaling pathways that collectively mitigate neuronal damage and cognitive decline [35,76,77]. Withanolides may inhibit BACE1 and enhance ADAM10, potentially reducing Aβ aggregation based on in vitro evidence [35]. Preclinical evidence also supports this: in PC-12 cells, withanamides A and C bind to Aβ25–35, preventing fibril formation and protecting against Aβ-induced cytotoxicity [76]. In transgenic APP/PS1 mice, the reduction in brain Aβ monomer levels occurred after oral administration of a semi-purified root extract, ultimately reversing behavioral deficits [35,77]. Similarly, in mice that were Aβ-injected, withanoside IV improved memory and prevented neuronal loss by attenuating Aβ (25–35)-induced neurodegeneration [35]. Data from a 2005 study by Kuboyama et al., (2005) displays Aβ(25–35)-induced memory-deficient mice and cultured rat cortical neurons significantly restoring axons and dendrites [78]. Tau pathology is indirectly addressed, as withaferin A inhibits tau accumulation in models of neurodegeneration, preserving microtubule stability and axonal transport [77].

Withaferin A and withanolide A mitigate inflammatory cascades and ROS by blocking NF-κB signaling, particularly through targeting IKKβ to prevent the release of key pro-inflammatory mediators such as TNF-α, IL-1β, IL-6, and MCP-1, and limiting nitric oxide buildup. At the same time, they stimulate Nrf2 activation to elevate defensive enzymes like superoxide dismutase (SOD), catalase, and glutathione peroxidase (GPx) [9,35,76,77]. In Wistar rats, oral Ashwagandha extract suppresses AChE activity and Aβ formation, leading to reduced cytokine expression and the mitigation of cognitive decline [35]. Preclinical findings indicate that Ashwagandha restores oxidative balance [35]. In SH-SY5Y cells, treatment with aqueous root extract (50–200 µg/mL) decreases ROS production and lipid peroxidation while enhancing GSH levels [35]. These effects converge, as reduced Aβ aggregation suppresses microglial and inflammasome activation, protecting against chronic neuroinflammation [9,76].

The plant enhances neurotransmission and synaptic plasticity by inhibiting AChE and upregulating choline acetyltransferase (ChAT), which increases acetylcholine availability. Furthermore, it modulates GABA and 5-HT receptors, contributing to broader neuromodulatory effects [9,35,76,77,78,79]. Withanolide A promotes BDNF and CREB activation, supporting synaptic plasticity, dendrite outgrowth, and neurogenesis [9,77,78,79]. In 5XFAD mice, intraperitoneal administration of sominone (a metabolite of withanoside IV) at 10 µmol/kg for 9 days increases synaptic density and improves cognitive function but has no effect on amyloid concentrations and microglial activation [35]. These effects protect synaptic structures, with preclinical outcomes showing restored long-term potentiation in hippocampal slices from AD models [9,77].

Gut–brain axis dysregulation is addressed, as Ashwagandha may modulate microbiota in neurodegenerative contexts. Withanoside IV undergoes deglycosylation by intestinal bacteria to form sominone, which is detectable in serum following oral administration and is capable of crossing the blood–brain barrier (BBB). Additionally, withanamides have demonstrated BBB permeability in vivo [35,76].

*Curcuma longa (Turmeric):* Turmeric, which harbors curcumin and its demethylated derivatives (demethoxycurcumin and bisdemethoxycurcumin), contains the key constituents in this plant for mitigating AD [81]. These compounds demonstrate promising efficacy in preclinical trials but are limited by poor bioavailability; therefore, enhanced delivery approaches are necessary [13,22,23,24,25,63,71,72,74]. Studies demonstrate that curcumin limits Aβ formation by downregulating the expression and activity of GSK-3β and CDK5, enzymes that drive β-secretase-dependent APP processing, thereby reducing Aβ generation and fibril assembly [13,20,63,71,72,73,74]. Narasingappa et al., (2012) established that ADAM10 activity is induced by curcumin’s conjugates [73]. In addition, curcumin alleviates AD symptoms by inhibiting inflammation and oxidative stress [13]. Preclinical studies show positive results on Aβ-induced cognitive decline; however, the results of clinical studies are not consistent, most likely due to the limited number of studies [81]. Das et al., (2019) demonstrated GSK-3β inhibition in scopolamine-induced AD rats, which addresses tau pathology [91]. By activating AMPK, curcumin mitigates neuroinflammation and oxidative stress while enhancing cellular energy balance. It neutralizes ROS through direct scavenging and suppresses NF-κB signaling, reducing the production of pro-inflammatory cytokines such as IL-1β, TNF-α, and IL-6 [13]. These effects attenuate oxidation and cytokine elevation in AD models [13]. These mechanisms synergize with amyloid reduction, as decreased Aβ mitigates further inflammatory activation [13,81].

Curcumin may improve gut–brain axis dysregulation and modulate microbiota in neurodegenerative disorders, as demonstrated by Shen et al., (2017), who found that curcumin effectively influences microbiota composition, decreasing Prevotellaceae significantly and increasing Bacteroidaceae and Rikenellaceae significantly, potentially counteracting AD progression [92].

*Ginkgo biloba:* Containing terpenoids like ginkgolides (A, B, C, and J) and bilobalide, along with flavonoids, stabilize mitochondrial membranes and enhance cerebral blood flow while also reducing Aβ-induced neurotoxicity and improving cognition in transgenic mouse models [34,36,39]. In a study conducted by Zhi-Xing Yao et al., (2004), ginkgolides and bilobalide inhibit amyloid formation by reducing free cholesterol levels [93], which alters APP processing to limit BACE1 activity, thereby preventing Aβ-induced membrane damage and aggregation [36]. Nowak et al., (2021) show that in both in vivo and in vitro administration of *Ginkgo biloba* extract, EGb 761, reduces Aβ levels, inflammation markers, activated microglia, and cytokines, all while improving cognitive function [34]. Furthermore, EGb 761 was administered in vivo at 100 mg/kg in mice, which—with the cellular senescent cells induced from nitrozation stress—effectively mitigated the induced stress [34]. These effects interconnect, with cholesterol modulation enhancing Aβ clearance and reducing neurotoxicity [36,39]. Ginkgolides and bilobalide also boost the expression of antioxidant enzymes such as SOD and catalase, effectively reducing lipid peroxidation [39]. This effect further aligns with amyloid reduction, as lower Aβ levels reduce glial priming—again counteracting the multifactorial progression of AD [34,39]. Ginkgolides also act as AchE inhibitors, which increase acetylcholine availability and activate BDNF/CREB pathways to promote long-term potentiation, aiding against cognitive decline [34,36]. Further explained by Singh et al., (2019), *Ginkgo biloba* increases cerebral blood flow while reducing clotting, and can help with fatigue and deficits in attention [36]. As for microbiota dysregulation, a study conducted by Yu et al., (2023) [94] shows that within in vivo models, 6-month-old male APP/PS1 transgenic mice that were treated daily with EGb (100 mg/kg) for 2 months exhibited gut microbiota remodeling, including a reduced Firmicutes/Bacteroidetes ratio, enriched Bacteroidetes/Uroviricota/Streptophyta/Spirochaetes, and elevated *Bifidobacterium pseudolongum* and *Limosilactobacillus reuteri*; these changes ultimately improved memory and learning while normalizing dysbiosis [80,94].

*Bacopa monnieri:* *Bacopa monnieri*, enriched with terpenoid saponins such as bacosides A and B, disrupts Aβ fibrillation and enhances synaptic plasticity via CREB activation, reversing memory deficits in scopolamine-induced AD rat models [37,40,41,49,80]. Satyam Sangeet and Arshad Khan investigated phytochemicals from *Bacopa monnieri* and found that there is a very unique inhibitory mechanism of the enzyme BACE1, leading to suppression of amyloid formation [37,40]. Ria et al., (2015) demonstrated in their study the reversal of spatial memory loss in a scopolamine-induced AD rat model, where rats were orally administered CDRI-08, an extract from *Bacopa monnieri*, at a dose of 200 mg/kg for 7 days [95]. Sushma et al., (2023) demonstrated the therapeutic effects of bacopa by treating Wistar rats that were injected with amyloid-β_42_, resulting in a GSK-3β interaction that leads to reducing hyperphosphorylation and preserving microtubule function [96]. Extensive research shows that bacopa mitigates ROS and suppresses pro-inflammatory cytokines like IL-6 and TNF-α while enhancing the expression of antioxidant enzymes such as SOD and catalase [37,49]. Kapoor et al., (2009) demonstrated a treatment with bacopa extract once a day for 15 days in streptozotocin-induced diabetic rats with symptoms similar to those of AD; the treatment showed a normalization of lipid peroxidation and restoration of glutathione levels, reducing mitochondrial dysfunction and energy deficits [97]. This synergizes with amyloid inhibition, as lower Aβ mitigates glial priming and oxidative burden [37]. Bacosides A and B inhibit AChE—increasing acetylcholine availability essential for cognitive signaling—and activate CREB signaling pathways to upregulate BDNF expression, promoting neurogenesis and long-term potentiation, critical for memory consolidation [37,40,41,49]. Moreover, Rastogi et al., (2012) showed how treatment with 200 mg/kg bacosides in a healthy aging female rat model reversed senile AD through a reduction of lipofuscin aggregation in neurons and reduced cholinergic degradation [98]. In PC-12 cells, bacoside A supports dendrite outgrowth and protects neurons from oxidative and inflammatory damage [37,49]. These effects protect against synaptic loss, with preclinical outcomes showing restored memory consolidation in maze tasks [40,41]. Gut–brain axis dysregulation may be mitigated by medicinal herbs such as Bacopa, which modulate microbiota in neurodegenerative disease [80]. In vivo studies of fecal cultures showed that Bacopa altered the microbiota significantly, increasing bacteria that produce butyrate, such as *Bacteroides xylanolyticus*, *B. uniformis*, and *Butyrivibrio crossotus*, and enhancing butyrate levels in the lumen [80]. Bacosides exhibit low absorption, often requiring microbial hydrolysis into active aglycones to enhance BBB penetration [10].

*Panax ginseng:* *Panax ginseng* contains triterpenoid saponins known as ginsenosides, like Rb1, Rg1, and Rd, which promote Aβ clearance and reduce tau phosphorylation while modulating the gut–brain axis via microbial metabolites like compound K [7,44,45,46,47]. A study carried out by Han et al., (2019) discovered cognitive improvements in APP/PS1 transgenic mice that were treated with minor ginsenoside F1 (20 mg/kg orally daily for 8 weeks), measured using a Y-maze; furthermore, immunostaining was performed to measure Aβ plaques and, interestingly, a reduction was found in the cortex but not the hippocampus [46]. However, we see evidence that ginseng extract can attenuate Aβ in the hippocampus in other in vivo studies [45]. Furthermore, tau pathology is thought to be addressed via PI3K/Akt signaling activation, which inhibits kinases like GSK-3β, shown by in vivo reduction of tau protein hyperphosphorylation, thereby preventing NFT formation [44,45,46,47]. These effects are interrelated, as decreased Aβ limits tau kinase activation, which in turn interrupts the pathological cycle [10,46]. Ginsenosides Rb1 and Rg1 aid in the suppression of prolonged microglial activation through the inhibition of LPS-induced response and NF-κB signaling; in turn, this reduces pro-inflammatory cytokines IL-1β, TNF-α, and IL-6. Collectively, this reduces cascades found in AD models by restoring SOD and GPx activity by Nrf2 activation, enhancing phagocytosis of Aβ, and preserving mitochondrial function [44,45]. Wang et al., (2011) showed in a rat model of AD that ginsenoside Rb1 effectively reduces oxidative stress and neuroinflammation [47]. This links to amyloid and tau pathologies since reducing aggregates and phosphorylation both attenuate inflammatory and oxidative amplifiers [45]. In AD models, mice display a reduction in acetylcholine synthesis, causing some of the reduction in cognitive ability—ginseng was able to increase acetylcholine production within in vitro and in vivo models [44,45]. Gintonin, when orally administered in mice, promoted BDNF expression, which promotes neurogenesis, dendritic outgrowth, and long-term potentiation, attenuating synaptic loss [44,45]. In multiple rodent models of neurodegeneration, we can see that ginseng extracts have neuroprotective and cognitive improvement effects [44,46,47]. Gut–brain axis dysregulation is improved through compound K, which is a metabolite product from ginsenosides Rb1 and Rg1 as it is transformed by gut microbiota, restoring microbial balance and elevating hippocampal serotonin levels [44,80,84]. Zhang et al., (2023) found increases in SCFA production in dysbiosis models caused by an increase in beneficial bacteria, in turn reducing systemic inflammation and modulating vagal signaling to attenuate CNS amyloid pathology and BBB permeability [84,99]. Bioavailability is low in rodents, however, due to the gut flora converting ginseng into metabolites capable of crossing the BBB, positive effects remain [10,84].

*Crocus sativus (Saffron):* *Crocus sativus* is commonly known as saffron, which contains polyphenolic compounds such as crocin and safranal. Preclinical evidence suggests its potential in reducing Aβ aggregation and tau pathology while demonstrating improved learning and memory in AD mouse models [14,50,51,80]. Preclinical evidence suggests that the attenuation of these pathologies occur through enzyme inhibition, antioxidant activation, and microbiota-mediated interaction; however, its bioavailability remains dependent heavily on gut microbial transformation [10,14,51,80]. AD models suggest that crocin and safranal inhibit amyloid formation by directly binding to Aβ peptides to inhibit fibril formation, and by enhancing clearance mechanisms through the aforementioned upregulation of enzymes like neprilysin [14,50,51]. Behavioral tests on AD models show evidence that crocin improves learning and memory, and it is suggested that the reduced Aβ burden is central to this [14,51]. A reduction in tau pathology is shown through in vivo experiments, and while the exact mechanism is unknown, it is postulated by Hatziagapiou et al., (2019) that it is related to the partial negative charge of the carbonyl group in corcin [51]. Saffron is rich in antioxidants, which leads to a reduction in ROS; in turn, this reduction leads to attenuating NF-κB signaling, which reduces the release of pro-inflammatory cytokines—as a result, dampening microglial overactivation and preserving neuronal integrity in AD models [14,51]. Concurrently, studies show Nrf2 pathway activation with saffron, which boosts antioxidant enzyme expression like SOD and catalase that further neutralize ROS [14,51]. AChE inhibition by saffron was demonstrated on ROT-treated flies, where AChE was elevated and then mitigated with saffron treatment, leading to elevated acetylcholine levels; other studies suggest the activation of BDNF and CREB signaling pathways—promoting dendritic stability and neurogenesis—by saffron [51,100]. In terms of the gut–brain axis component, we see two contradictory mouse studies, where one shows an increase in SCFA, which are known to help with AD, while another study shows a disturbance of the microbiota, which led to an exacerbation of inflammation [80].

*Camellia sinensis (Green Tea):* *Camellia sinensis* or green tea is abundant in polyphenols like epigallocatechin-3-gallate (EGCG) and theanine [6,12,15,18,19,70]. EGCG and theanine are the key polyphenols, and as amino acid derivatives from *Camellia sinensis*, they inhibit amyloid formation by suppressing BACE1 activity while binding directly to regions of Aβ [15,18,19]. In a streptozotocin-induced dementia rat model where rats were treated with EGCG at 10 mg/kg/day for 4 weeks, cognitive deficits were reversed, as measured by the Morris Water Maze [18]. Furthermore, the results suggest that EGCG inhibits BACE1, which in turn reduces Aβ production and lipid peroxidation [18]. EGCG and theanine mitigate neuroinflammation by suppressing the production of pro-inflammatory cytokines, including IL-1β and TNF-α, by directly neutralizing ROS, preventing neuronal oxidative damage, and restoring hippocampal redox balance [15,18]. Pairing this, catechins also mitigate tau pathology by inhibiting hyperphosphorylation through GSK-3β [15]. Studies indicate that EGCG activates the Nrf2 pathways to upregulate antioxidant enzymes such as SOD and GPx, aiding mitochondrial function [15,18,70]. EGCG targets cholinergic dysfunction by reducing AChE activity as part of its multi-target profile, as indicated in the in vivo study conducted on a streptozotocin rat model [18]. Additionally, in a population that had a higher intake of epicatechin, a lower risk of Parkinson’s disease was observed because of the increase in CERB upregulation, which promotes dendritic stability and long-term potentiation [15,19]. In another study on the gut–brain axis, ovariectomized mice that were fed a high-fat diet meant to put the mice at risk for AD were administered 45 mg/kg of EGCG, which significantly restored spatial and episodic memory deficits that were observed in behavioral assays; this stems from EGCG’s remodeling of the induced gut dysbiosis in the mice. 16S rRNA sequencing confirmed the reverse of microbiota loss, the increase in Prevotella, and the suppression of Bifidobacteriales [101].

*Salvia officinalis:* *Salvia officinalis*, also known as sage, contains polyphenols such as rosmarinic acid and carnosic acid [16,17,52]. Noguchi-Shinohara et al., (2020) show that, within hippocampal slices, rosmarinic and carnosic acids counteract amyloid formation by directly binding to Aβ peptides, disrupting fibril assembly and inhibiting oligomerization [16,17]. In AD rodent models, sage extract is shown to attenuate memory deficits in Aβ-injected rats, while also playing a beneficial role in tau pathology by preventing Aβ-induced hyperphosphorylation [16]. Together with the inhibition of Aβ aggregation, these mechanisms act to reduce downstream inflammatory and oxidative cascades [16,17]. Aside from preventing downstream oxidative cascades, rosmarinic and carnosic acids inhibit the COX-2 and NF-κB pathways, suppressing the release of pro-inflammatory cytokines such as TNF-α and IL-6, while activating Nrf2 signaling to enhance antioxidant defenses [16]. These mechanisms and compounds amplify ROS neutralization and protect against lipid peroxidation and mitochondrial dysfunction [16,52]. Rosmarinic and carnosic acids enhance BDNF expression to support synaptic resilience, as shown in a study where the compounds mitigated BDNF loss in AD mice, and in another study showing the inhibition of AChE [16]. Furthermore, rosmarinic acid protects against synaptic loss through anti-inflammatory effects [52,53]; additionally, sage extract has been shown to promote CREB signaling [53] in rodent models. Collectively, these actions elevate acetylcholine decreases caused by AChE, and support cortical function by upregulating BDNF and CREB [52].

*Melissa officinalis:* *Melissa officinalis* (lemon balm) contains the polyphenol rosmarinic acid, and as such, the effects are very similar to *Salvia officinalis* [17,54,55,56]. Unlike sage, which gains benefits from carnosic acid’s additional COX-2 inhibition and antioxidant effects for broader neuroprotection effects, lemon balm solely drives Aβ disruption and neuronal preservation through rosmarinic acid. As mentioned, rosmarinic acid significantly reduces toxic cluster formation and plaque deposition, mitigating neuronal damage, as demonstrated in vitro studies [17,56]. Preclinical evidence from in vitro models shows rosmarinic acid disrupting Aβ assembly and mitigating cytotoxicity in neuronal cultures [54]. In preclinical studies, it promotes Aβ clearance by reducing BACE mRNA expression in key areas like the hippocampus [56]. With the reduction in aggregates, the reduction in oxidative and inflammatory effects follows [54,56]. However, more directly, rosmarinic acid mitigates neuroinflammation and oxidative stress by neutralizing ROS, suppressing NF-κB signaling to reduce cytokine production, which also helps mitochondrial integrity to prevent dysfunction [54]. In vivo microglial cultures of rosmarinic acid reduce ROS and cytokine release, showing its anti-inflammatory properties [54]. Preclinical studies also demonstrate a reduction in lipid peroxidation, which aids the structural integrity of the cells, and potentially has synergy with a reduction in glial activation [54,56]. Rosmarinic acid enhances neurotransmission and synaptic plasticity by inhibiting AChE and modulating monoamine systems, such as serotonin and dopamine, to strengthen cholinergic signaling [54,55,56]; this leads to significant increases in acetylcholine and monoamine levels, which support cognitive function and memory retention [56]. In rodent models that were treated with scopolamine, Melissa extract improved both cognitive and behavioral responses [56]. Although direct gut–brain axis details are limited, the increases in monoamine modulation may indirectly influence the gut–brain axis, which would lead to a reduction in systemic contributions to AD pathology [80]. Bioavailability is limited by rapid metabolism due to the nature of these compounds, which indicates the need for formulations that enhance systemic availability and BBB permeability [10].

*Centella asiatica:* Featuring triterpenoids such as asiaticoside and madecassoside, these compounds have a multitude of effects, from enhancing synaptic density via BDNF and reducing Aβ plaque formation to improving cognition in 5XFAD transgenic mice, despite the issue in variable bioavailability [38,42,43,57]. Asiaticoside and madecassoside both inhibit Aβ aggregation, suggestive of binding to Aβ peptides, leading to a reduction of plaque buildup in AD models [43]. The compounds display anti-neuroinflammatory properties through the suppression of NF-κB, which reduces cytokines and leads to a reduction in microglial activation, further mitigating AD [43]. In preclinical trials, *Centella asiatica* water extract, rich in triterpenoids asiaticoside and madecassoside, was administered orally at 200 mg/kg, which did not significantly reduce hippocampal Aβ plaque load as measured by immunohistochemistry, yet still improved cognitive function [42]. Furthermore, complementary in vivo studies show that asiaticoside prevents early Aβ aggregation [43]. Asiaticoside and madecassoside further alleviate neuroinflammation and oxidative stress by directly neutralizing ROS and Nrf2 activation. Moreover, an in vivo study performed by Gary et al., (2018) demonstrated how canella asiatica improved twenty-month-old CB6F1 mice with behavioral, learning, memory, and executive functions, in addition to a Golgi analysis of spine density showing an increase in synaptic density [38,42,43,57]. Madecassoside treatment (30 mg/kg) for 60 days in aged mouse models normalized ROS levels and reduced lipid peroxidation, which aided in restoring mitochondrial function and energy homeostasis [38,43]. Additionally, preclinical neurodegenerative models show that the extract attenuates microglial hyperactivity, linking anti-inflammatory effects to preserved hippocampal structures [43,57]. Asiaticoside enhances cognitive function by increasing synaptic density, potentially by activating CREB and BDNF signaling to promote neurogenesis while inhibiting AChE to elevate acetylcholine levels [38,42,43,57]. Matthews et al., (2019) showed in 5XFAD mice that *Centella asiatica* water extract increases synaptic markers, such as synaptophysin and PSD-95, showing increases in pre- and post-synaptic density [38,42]. These effects collectively strengthen hippocampal plasticity, aiding cognitive function [38]. Nevertheless, bioavailability remains a limitation as it does in all plants since asiaticosides display low absorption in rodents, and often require microbial transformation to cross the BBB and generate active metabolites or chemical modification to improve bioavailability [57].

## 5. Multi-Target Approaches of Phytochemicals

**Figure 1:**
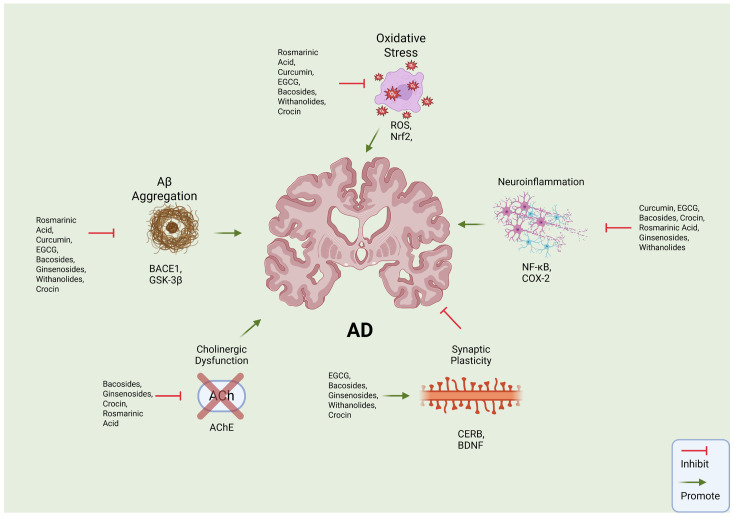
Neuroprotective mechanisms of phytochemicals targeting AD pathology. Schematic depicting key pathogenic pathways implicated in AD, including Aβ accumulation, oxidative stress, neuroinflammation, cholinergic dysfunction, and synaptic plasticity impairment. Phytochemicals exert protective effects through multiple approaches such as inhibiting Aβ aggregation by downregulating BACE1 and GSK-3β, reducing oxidative stress by modulating ROS and activating Nrf2, suppressing neuroinflammation through inhibition of NF-κB and COX-2 signaling, preventing cholinergic dysfunction by inhibiting AChE activity, and strengthening synaptic plasticity through the upregulation of CREB and BDNF pathways.

Multi-target approaches, also known as polypharmacology, integrate bioinformatics, protein interaction data, and chemical properties of compounds to interpret interactions across biological networks [69]. This method of approach is valuable for analyzing plant-derived compounds by revealing how they exert synergistic effects and modulate multiple pathways in multifactorial diseases such as AD, offering advantages over current single-target therapies in addressing AD [69]. For instance, Aβ aggregation increases oxidation, which leads to an increase in inflammation by the activation of NF-κB, followed by tau hyperphosphorylation [1]. GSK-3β under normal conditions adds phosphates to other proteins for normal function; however, in AD, the pathway is overactivated, leading to tau hyperphosphorylation—making it a valuable target to regulate [71]. Another key target is secretases because of their involvement with the production of amyloid by cleaving APP, leading to amyloid aggregation [5]. Another point of interest is Nrf2, when the cell is stimulated by oxidative stress; Nrf2 starts an antioxidant response leading to the attenuation of inflammation and oxidation [102,103]. In addition, CREB is linked to BDNF, which regulates cognitive function and promotes neuronal plasticity, and it is also suggested that ROS decrease the phosphorylation of CREB, decreasing its expression [102]. ACh is involved in neuroplasticity and aiding synaptic connections; in AD, cytokines can increase AChE activity, decreasing ACh, making AChE a therapeutic target [5]. Table 2 summarizes the key pathways and targets in AD by phytochemical compounds. Plant-derived compounds, as shown through the studies, interact with multiple AD pathways [10,71]. For example, curcumin from *Curcuma longa* inhibits GSK-3β and alleviates inflammation and oxidative stress caused by AD [13,71,81] while also influencing pathways such as PI3K-Akt, MAPK, and apoptosis signaling [75]. Furthermore, withanolides from *Withania somnifera* (Ashwagandha) modulate the HPA axis to reduce cortisol, activate Nrf2 to reduce oxidation, and inhibit AChE while promoting neurogenesis and anti-inflammation [35,77]. Another example is EGCG from *Camellia sinensis* (green tea), showing properties of BACE1 inhibition to reduce amyloid accumulation [19] and decreasing AChE activity, supporting cholinergic function while providing antioxidant benefits, etc. [18]. The multi-target nature of these phytochemicals is extremely advantageous for AD, as it addresses all the pathological cascades rather than each individual pathway with the aim of reducing the progression of AD. Figure 1 illustrates the neuroprotective mechanisms of phytochemicals targeting AD pathology.

## 6. Addressing Bioavailability and Delivery Science

The biggest challenge when treating with phytochemicals is their low bioavailability and BBB penetration; however, new vesicular delivery systems have been shown to enhance stability, BBB crossing, and efficacy in AD models [22].

*Liposomes:* Composed of phospholipids and cholesterol, forming bilayer vesicles, liposomes work by encapsulating hydrophilic/lipophilic phytochemicals, protecting them from degradation while promoting endocytosis. Although they offer advantages, they are limited by their short circulation time and stability issues [22].

*Niosomes:* Non-ionic surfactants and cholesterol form the vesicular structure for niosomes. They function through controlled release, while protecting the encapsulated compounds from degradation and improving oral and even transdermal bioavailability via lipid fusion with cell membranes. Major advantages include their cost-effectiveness and stability [27]. Preclinical studies have demonstrated that curcumin-loaded niosomes increased object recognition and T-maze test results in AD rats better than curcumin alone, and also had more of an impact on NF-κB expression [28].

*Transfersomes:* Being composed of phospholipids and edge activators, transfersomes form highly deformable and flexible vesicles. They function by penetrating the skin through intracellular junctions that enable systemic and brain delivery. Their advantages include non-invasiveness and high deformability. Preclinical in vivo studies have demonstrated that insulin-loaded transfersomes show higher retention and targeting compared to free insulin [24,27].

*Ethosomes:* Composed of phospholipids with 20–50% ethanol content, ethosomes form flexible vesicles. They work through ethanol’s ability to increase lipid permeability for deeper transdermal penetration, enhancing bioavailability. Due to electrostatic repulsion and steric stabilization, the ethosomes more effectively entrap while maintaining a smaller particle size than liposomes [27]. Shi et al., (2012) have highlighted that ethosomes loaded with an antioxidant known as ligustrazine phosphate administered transdermally for treatment in AD rats improved performance in the Morris Water Maze, showing that drug deposition was much higher with ethosomes than aqueous solution [104].

*Bilosomes:* These are liposomes but with the addition of bile salts, with the purpose of mitigating some of the drawbacks of liposomes, like protection in the harsh environments of the gastrointestinal tract. They display enhanced bioavailability due to the bile salt interactions [26]. In AD mice, curcumin + miR-101 bilosomes decreased Aβ42 levels and inflammation, while RES-loaded bilosomes reduced activated IbA1/++ microglial cells, alleviating neuroinflammation [26]. Similarly, bilosome-enhanced celecoxib shows better neuroprotective effects in AD models compared to free celecoxib [29]. Elsheikh et al., (2022) showed a similar result with luteolin bilosomes, which were more effective than luteolin suspension in AD mice [30].

*Solid Lipid Nanoparticles (SLNs):* Composed of lipids similar to those of liposomes; however, the differences lie in the interaction of the cargo that is being delivered. The heads of the phospholipids individually interact with cargo, and as a result, there is sustained drug release and better BBB penetration. Offering high loading and stability, a major limitation is drug expulsion during storage and mobility of the particles [32]. In an AD rat model, Khishvand et al., (2024) have shown that resveratrol-loaded SLNs improved Morris Water Maze performance, improved memory, lowered lipid peroxidase, and elevated GSH levels better than free resveratrol [32].

*Nanostructured Lipid Carriers (NLCs).* Commonly referred to as second-generation SLNs, NLCs are composed of blends of solid and liquid lipids, forming imperfect crystal structures. They provide improved drug entrapment, stability, biodegradability, and permeability. An in vitro study by Agrawal et al., (2021) demonstrated that curcumin-loaded NLCs showed prolonged release better than free curcumin, improving the stability and therapeutic potential for AD treatment [25].

## 7. Bridging Preclinical and Clinical Evidence

Bridging the gap between preclinical and clinical evidence is crucial for validating the therapeutic potential of plant-derived compounds in AD management, since direct translations from preclinical to clinical do not always occur, which are better known as translational issues. Preclinical investigations, typically in cellular and animal models, provide mechanistic insights into neuroprotective effects against AD pathologies; meanwhile, clinical trials evaluate the safety and efficacy of outcomes in human populations [11]. Table 3 organizes key preclinical and clinical findings for selected plants, identifying gaps that underscore the need for large-scale studies to facilitate clinical adoption [10,11]. Nonetheless, most of the compounds remain in the preclinical stages due to phytochemical research facing further challenges like extract variability, lack of standardization, and model discrepancies like in vitro showing more success than in vivo due to the bioavailability and stability of phytochemicals. As such, there is a major inconsistency seen in clinical trials; the success of trials may be contributed to smaller sample sizes and poor cognitive test designs. In addition, it is critical for reproducibility to know how the plants in question are sourced, the composition of soil the plants grew in, the time of year, exposure to the sun, and the temperature of the surrounding area. Also, it is important to know when the plants were harvested and how they were processed when it came to making the crude or purified extracts and their resulting concentrations from the process. Furthermore, with a lack of phase II/III clinical trials, it is difficult to conclude how well these compounds work in AD—issues like small sample size and short durations remain and are critical when determining efficacy. Further compounding these issues, many clinical trials are in healthy individuals, and clinical trials on patients with AD are limited, further hindering translational data. In addition, cognitive assessments like the Mini-Mental State Examination (MMSE) and Alzheimer’s Disease Assessment Scale–Cognitive Subscale (ADAS-Cog) may not detect subtle differences to disease modification or differentiate between symptomatic relief. Variability in the test selection and administration of tests across studies further hinder comparisons. Addressing these through standardized preparations, increasing the quantity of phase II/III trials, and incorporating more biomarkers like CSF Aβ/tau levels paired with neuroimaging is essential for advancing phytochemicals in their clinical application.

## 8. Conclusions

AD poses a global challenge with its multifactorial pathology driving progressive cognitive decline. This review highlights the potential of plant-derived bioactive compounds rooted in traditional medicine as multi-target therapies capable of addressing the multifactorial aspect of AD. Polyphenols, terpenoids, and alkaloids demonstrate neuroprotective effects in preclinical models, and emerging cognitive benefits in early AD and mild cognitive impairment are still modest due to small sample sizes and the lack of better ways to measure cognitive gains or decline. Their ability to address multiple AD pathways offers a compelling alternative to single-target drugs, further enhancing therapeutic potential. Poor bioavailability, BBB permeability, and volatility seem to be the main hurdles for these compounds and molecules; however, these are challenges that can be overcome with advancements in delivery systems. The best results for the compounds and molecules are seen within in vitro experiments, where there is no exposure to the harsh digestive environment and no BBB to overcome. More research must prioritize large-scale clinical trials to validate efficacy across diverse populations. By integrating these phytochemicals into preventive and therapeutic strategies, there is the potential to mitigate the projected rapid increase in the population with AD.

## Figures and Tables

**Table 3:** Translational evidence of phytochemicals from preclinical mechanisms to clinical outcomes in AD.

Plant	Preclinical Key Findings	Clinical Key Findings
*Withania somnifera*	Dendrite outgrowth and regeneration (in vitro WL-A 1 μM for 7 days) memory recovery (in vivo 10 μM/kg daily for 13 days) [78].	Memory improvement in MCI (*n* = 50, RCT, 300 mg twice daily, 8 weeks) [9].
*Curcuma longa*	Improving cognitive/spatial memory while reducing cytokines and increasing SOD in amyloid-β1–42-injected mice (150 mg/kg daily 10 days) [13].	Cognitive gains (*n* = 79, RCT, 80 mg orally, once daily, 12 weeks) [105]. No observable difference between placebo and treatment group (*n* = 96, RCT, 12 months, 1500 mg daily) [106]. Improved cognitive and locomotive function in AD (*n* = 48, RCT, 800 mg daily for 6 months) [107]. Improved memory and attention (*n* = 40, RCT, 180 mg daily for 18 months) [108].
*Ginkgo biloba*	Decreased Aβ production and attenuated synaptic loss in APP mice (240 mg daily for 5 months) [39].	Improved symptoms in mild dementia (*n* = 782, meta-analysis of RCT, 240 mg) [64].
*Bacopa monnieri*	Improved memory and learning in streptozotocin Wistar rats (30 mg/kg orally 2 weeks) [37].	Memory enhancement in healthy adults (systematic review of 6 RCTs, 3 of which used 450 mg daily) [65].
*Panax ginseng*	Spatial working memory improvements in APP/PS1 mice (20 mg/kg daily orally of F1 for 8 weeks) [46]. Reversed neuroinflammation in Aβ1–42-injected rats treated with Rb1 [47].	Cognitive improvement in AD trials (*n* = 97, RCT, 4.5 g daily for 12 weeks) [109].
*Crocus sativus*	Decreased neuroinflammation (*n* = 30 rats, 20 mg/kg for 7 days) [110]. Increased antioxidants and decreased lipid peroxidation (MCAO rats, 72.5, 145 mg/kg for 4 weeks) [111].	Significantly improves cognition (*n* = 54, RCT, 30 mg daily, 22 weeks; *n* = 46, RCT, 30 mg daily, 16 weeks; *n* = 68, RCT, 30 mg daily, 48 weeks; *n* = 35, RCT, 125 mg daily, 48 weeks) [66].
*Camellia sinensis*	Cognitive improvement, decreased Aβ and tau (APPSw mice, 20 mg/kg for 60 days intraperitoneal injection, 50 mg/kg for 6 months orally) [112].	Lowers risk of cognitive disorder (meta-analysis of *n* = 21,444, 6 cohort, *n* = 6249, 3 case-control, *n* = 20,742, 8 cross-sectional studies) [21].
*Salvia officinalis*	Lowers AChE activity in mice (300 mg/kg for 7 days) [113]	Significant improvement in cognition (*n* = 42, RCT) [114].
*Melissa officinalis*	Aβ reduction in AD mice (Tg2576) for 10 months from the age of 5 months orally [115].	Reduced agitation and improved cognitive function in patients with AD (*n* = 42, RCT, 4 months) [67].
*Centella asiatica*	Increased synaptic density and cognition (20-month-old CB6F1 mice, 2 mg/mL in drinking water for 2 weeks) [38].	No difference in cognitive function (meta-analysis of 11 RCTs) [68].

## Data Availability

No new data were created or analyzed in this study.
